# Gender disparity in cases enrolled in clinical trials of visceral leishmaniasis: A systematic review and meta-analysis

**DOI:** 10.1371/journal.pntd.0009204

**Published:** 2021-03-16

**Authors:** Prabin Dahal, Sauman Singh-Phulgenda, Piero L. Olliaro, Philippe J. Guerin

**Affiliations:** 1 Infectious Diseases Data Observatory–IDDO, Oxford, United Kingdom; 2 Centre for Tropical Medicine and Global Health, Nuffield Department of Medicine, University of Oxford, Oxford, United Kingdom; Ohio State University, UNITED STATES

## Abstract

**Background:**

A higher caseload of visceral leishmaniasis (VL) has been observed among males in community-based surveys. We carried out this review to investigate how the observed disparity in gender distribution is reflected in clinical trials of antileishmanial therapies.

**Methods:**

We identified relevant studies by searching a database of all published clinical trials in VL from 1980 through 2019 indexed in the Infectious Diseases Data Observatory (IDDO) VL clinical trials library. The proportion of male participants enrolled in studies eligible for inclusion in this review were extracted and combined using random effects meta-analysis of proportion. Results were expressed as percentages and presented with respective 95% confidence intervals (95% CIs). Heterogeneity was quantified using I^2^ statistics and sub-group meta-analyses were carried out to explore the sources of heterogeneity.

**Results:**

We identified 135 published studies (1980–2019; 32,177 patients) with 68.0% [95% CI: 65.9%–70.0%; I^2^ = 92.6%] of the enrolled participants being males. The corresponding estimates were 67.6% [95% CI: 65.5%–69.7%; n = 91 trials; I^2^ = 90.5%; 24,218 patients] in studies conducted in the Indian sub-continent and 74.1% [95% CI: 68.4%–79.1%; n = 24 trials; I^2^ = 94.4%; 6,716 patients] in studies from Eastern Africa. The proportion of male participants was 57.9% [95% CI: 54.2%–61.5%] in studies enrolling children aged <15 years, 78.2% [95% CI: 66.0%–86.9%] in studies that enrolled adults (≥15 years), and 68.1% [95% CI: 65.9%–70.0%] in studies that enrolled patients of all ages. There was a trend for decreased proportions of males enrolled over time: 77.1% [95% CI: 70.2%–82.8%; 1356 patients] in studies published prior to the 1990s whereas 64.3% [95% CI: 60.3%–68.2%; 15,611 patients] in studies published on or after 2010. In studies that allowed the inclusion of patients with HIV co-infections, 76.5% [95% CI: 63.8%–85.9%; 5,123 patients] were males and the corresponding estimate was 64.0% [95% CI: 61.4%–66.5% 17,500 patients] in studies which excluded patients with HIV co-infections.

**Conclusions:**

Two-thirds of the participants enrolled in clinical studies in VL conducted in the past 40 years were males, though the imbalance was less in children and in more recent trials. VL treatment guidelines are informed by the knowledge of treatment outcomes from a population that is heavily skewed towards adult males. Investigators planning future studies should consider this fact and ensure approaches for more gender-balanced inclusion.

## Author summary

Visceral Leishmaniasis (VL) is a neglected tropical disease with an estimated incidence of 50,000 to 90,000 cases in 2019 but the case burden used to be much higher. This drop is mostly the result of the ongoing elimination campaign in the Indian sub-continent that started in 2005. A preponderance of male cases has been observed in community-based surveys carried out in VL endemic regions and also in some clinical trials. We did a systematic review of all published clinical trials (1980–2019) and carried out a random effects meta-analysis estimating the proportion of males enrolled in these studies. We found that overall between two-thirds and three-fourths were males, with substantial heterogeneity in the estimates across and within regions and the imbalance was less pronounced in studies enrolling children. Despite a trend for decreasing proportion of males enrolled over time, there were still 1.8 times as many males than females even in more recent trials. Our review suggests that antileishmanial therapeutic efficacy guidelines are informed by knowledge derived from a study population that is heavily skewed towards adult males, overwhelmingly from high-burden countries of the Indian sub-continent and Eastern Africa, though with some notable regional variations and substantial heterogeneity. This situation has led to a substantial lesser knowledge in optimal treatment strategies for female patients.

## Introduction

The epidemic of visceral leishmaniasis (VL) in the tea gardens of Assam, India, in the late 1800s equally affected both males and females [1]. At the turn of the century, male predominance in case burden was reported in other states of India as well as in China and Sudan while the disease was observed mostly among females in Italy and equally among males and females in Malta [2–5]. In the 1940s, Lionel E. Napier, a British physician who spent a large part of his career in India, attributed the observed asymmetrical caseload to “errors in selection” [6]. By errors, Napier probably meant that males were more likely to seek care and thus such behaviour led to a larger number of reported cases among males. In the 1950s, after correcting for selection error described by Napier, Chatterjee reported an adjusted ratio of male to female cases close to parity (adjusted: 53.0% males and 47.0% females; unadjusted: 61.4% males and 38.6%) at an in-patient hospital ward in Bihar [7]. Several observational reports published in the latter half of the century and at the turn of the millennium continue to report male predominance in caseload [8–13].

Two major hypotheses have been posited as a potential explanation for these differences: the behavioural hypothesis and the physiological hypothesis [14]. The former posits that a higher rate of infection observed among males emerges as a result of higher risk of exposure to the pathogens driven by socially-constructed roles leading to different cultural, environmental and lifestyle behaviours, and different access to care. The latter posits that the underlying biological differences between males and females at the molecular and cellular level could lead to the sex bias [15, 16].

The behavioural hypothesis has been long used as a basis for explaining the higher caseload observed among males in VL studies [4, 7, 9, 17]. One of the earliest explanations supporting this hypothesis was that of increased itinerancy among males making them more vulnerable to the disease acquisition [4, 18]. The occupational association of males with agriculture and construction brought them into frequent contact with the breeding sites of sand-flies [19]. Gender differences in treatment-seeking behaviour and differential access to healthcare could further drive the observed disparity [20–23]. Studies conducted in the past two decades have continued to point out cultural barriers, behavioural differences, or poor access to health care as factors leading to delayed clinical presentation among women [9, 17, 21, 24]. This observation remains contemporary, as reported in a recent study showing that India’s poorest women tend to ignore VL merely as a fever and do not seek hospital care [24].

Support for the physiological hypothesis comes mostly from experimental studies. Two studies in mice infected with *Leishmania* spp. found that disease severity is exacerbated among females who were administered testosterone hormone [15, 16]. Animal studies have demonstrated that males usually develop higher parasitaemia than females for leishmaniasis [25, 26]. Similarly, rodents treated with oestrogen hormone have demonstrated elevated antibody responses to T-cell-dependent and independent antigens [27]. Recently, Cloots et al (2020) conducted a large study in the Indian sub-continent to delineate the contribution of gender influenced socio-cultural behaviour from biological determinants [28]. By mitigating the impact of gender-related factors, the authors concluded that male sex is a risk factor for VL due to biological reasons and not only as a socio-cultural determinant.

A higher proportion of male enrolments has also been observed in several clinical trials in VL, generally conducted in endemic settings [9, 29–33]. We conducted a systematic review of all published clinical trials to address the following objectives: (i) to quantify the proportion of males enrolled, and (ii) to investigate if the observed differences in caseloads are explained by geographical region, study design or patient characteristics.

## Material and methods

### Literature search and eligibility criteria

We searched all the articles indexed in the open-access Infectious Diseases Data Observatory (IDDO) visceral leishmaniasis clinical trials library [34]. The IDDO VL clinical trials library is based on a living systematic review and the database is continually updated every six months in accordance with the Preferred Reporting Items for Systematic-Reviews and Meta-Analyses (PRISMA) guidelines (S1 Text). The trial library indexes publications identified from the following databases: PubMed, Embase, Scopus, Web of Science, Cochrane, clinicaltrials.gov, WHO ICTRP, Global Index Medicus, IMEMR, IMSEAR, and LILACS. For this current review, the search includes all clinical trials published between 1^st^ of Jan 1980 and 2^nd^ of May 2019. Details of the search strategy adopted have been previously described elsewhere [35, 36]. Studies indexed in the IDDO VL library were eligible for inclusion in this review if they met the following criteria: i) describing therapeutic efficacy of an antileishmanial regimen, and ii) gender not a part of exclusion criteria. Studies only among pregnant women or only among male participants were excluded. Studies that did not report the number of males and females participants were also excluded. The review was not limited by language.

### Study selection and data extraction

Data on the following aspects of the included studies were extracted: study design, location, publication year, the age range of participants, the total number of participants enrolled including the number of male and female patients, inclusion of pregnant and lactating women and women of childbearing age, requirement to undertake pregnancy test, use of contraceptives during treatment and follow-up period, and inclusion of patients with HIV co-infections. In addition, the mean (or median) duration of illness at enrolment was extracted when reported. Since women of childbearing age are either excluded from VL trials or required to adopt contraceptive methods due to known embryotoxic and teratogenic risks associated with some study drugs (e.g. miltefosine), we also extracted the details of the drug regimens that was studied. If the exact number of male and female patients were not reported, the information was calculated based on reported percentages. The first reviewer (SSP) independently extracted data from all eligible records which was verified by the second reviewer (PD) on all publications. Any differences in the extracted information was resolved through consensus.

### Definitions

Patient populations were grouped into the following age categories: children (<15 years), older children and adults (≥15 years). If participants from each age group were reported, then they were grouped as participants of “all ages.” Countries were classified into sub-regions according to the United Nations designation of areas and regions [37]. Study drugs were classified into different FDA categories for usage in pregnant and lactating women: Category B (no evidence of risks to the mother-child pair), Category C (risk cannot be ruled out), Category D (positive evidence of risk)[38, 39].

### Statistical analyses

The proportion of male patients enrolled in the clinical studies were combined using random effects meta-analysis after applying logit transformation and using the inverse variance weighting method. Heterogeneity was assessed using I^2^ statistics which quantifies the proportion of total variability attributable to between-study differences [40]. The pooled estimates were presented together with the associated 95% confidence intervals (95% CIs). Sub-group meta-analyses were undertaken to explore potential sources of heterogeneity. Potential small-study effects were investigated using Egger’s test and trim and fill method was used to obtain a bias-adjusted estimate. All statistical analyses were carried out using R software [41].

### Risk of bias assessment

The assessment of the risk of bias in studies included was carried out by adapting the Cochrane Risk of Bias tool for randomised controlled trials. Since patient-centred outcome variables were not analysed in this review, we assessed the risk of bias in randomised studies based on three domains related to participant enrolment: random sequence generation, allocation concealment, and blinding of participants and personnel. Similarly, the risk of bias in non-randomised studies were assessed based on the following domains related to participant enrolment: bias due to confounding, bias in selection of participants, and blinding of participants and personnel. Two reviewers (PD and SSP) independently assessed the risk of bias on each article.

## Results

### Study selection

**Fig 1:**
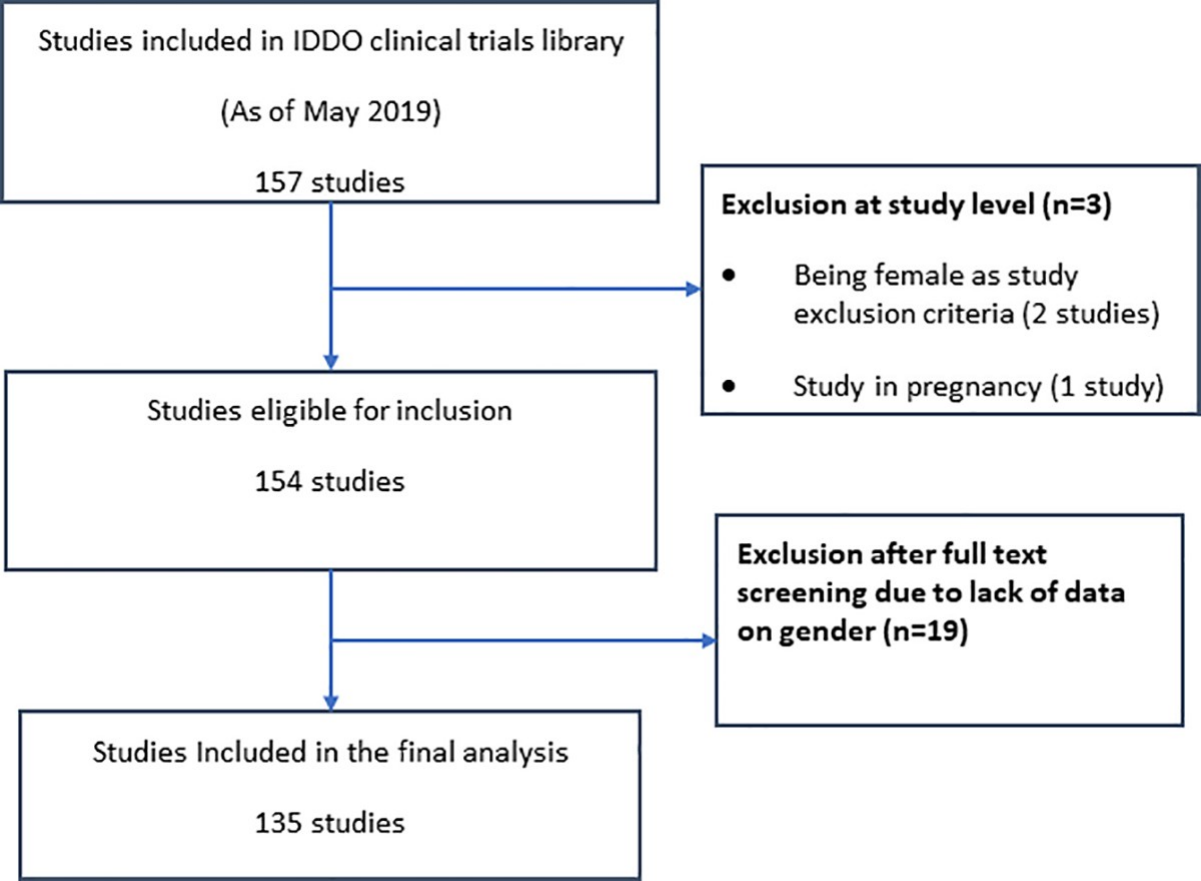
Preferred Reporting Items for Systematic Reviews and Meta-Analyses (PRISMA) flow diagram of screened publications.

The IDDO VL clinical trials library has indexed 157 therapeutic efficacy studies published between 1983 through 2019 [34]. Two studies that enrolled only male participants and one study in pregnant women were excluded, leaving 154 articles that were eligible for inclusion for this review. A further 19 trials were excluded after full-text screening as the number of patients for each gender status either were either not available or could not be reliably extracted, leading to a total of 135 studies included in this review (Fig 1). Of these, 91 studies (67.4%) were from the Indian sub-continent (1984–2019), 24 (17.8%) from Eastern Africa (1983–2019), 8 (5.9%) from the Mediterranean region (1994–2003), 7 (5.2%) from Southern America (1993–2017), 2 (1.5%) from Southern and Western Asia (Yemen and Iran) (2001–2009) and 3 (2.2%) were multi-regional (1996–2000). Fifty-three (39.3%) studies randomly allocated patients to treatment regimens, 71 (52.6%) were either single-armed studies or had non-randomised patient allocation and the randomisation status was not clear in 11 (8.1%). Pregnant or lactating women were excluded in 57 (42.2%) studies, included in 11 (8.2%), and their inclusion was unclear in 67 (49.6%) studies. Women of childbearing age were excluded in 1 (0.7%) study while their inclusion was conditional on agreement to practice contraception during treatment and ensuing follow-up period in 15 (11.1%) studies. Only 29 (21.5%) publications specified the phase of the trial. There were 23 (17.0%) Phase II-III studies, 6 (4.4%) Phase IV (post-registration) studies and the phase of the study was missing or not clear for remaining 106 (78.5%) studies.

### Overall gender distribution

**Fig 2:**
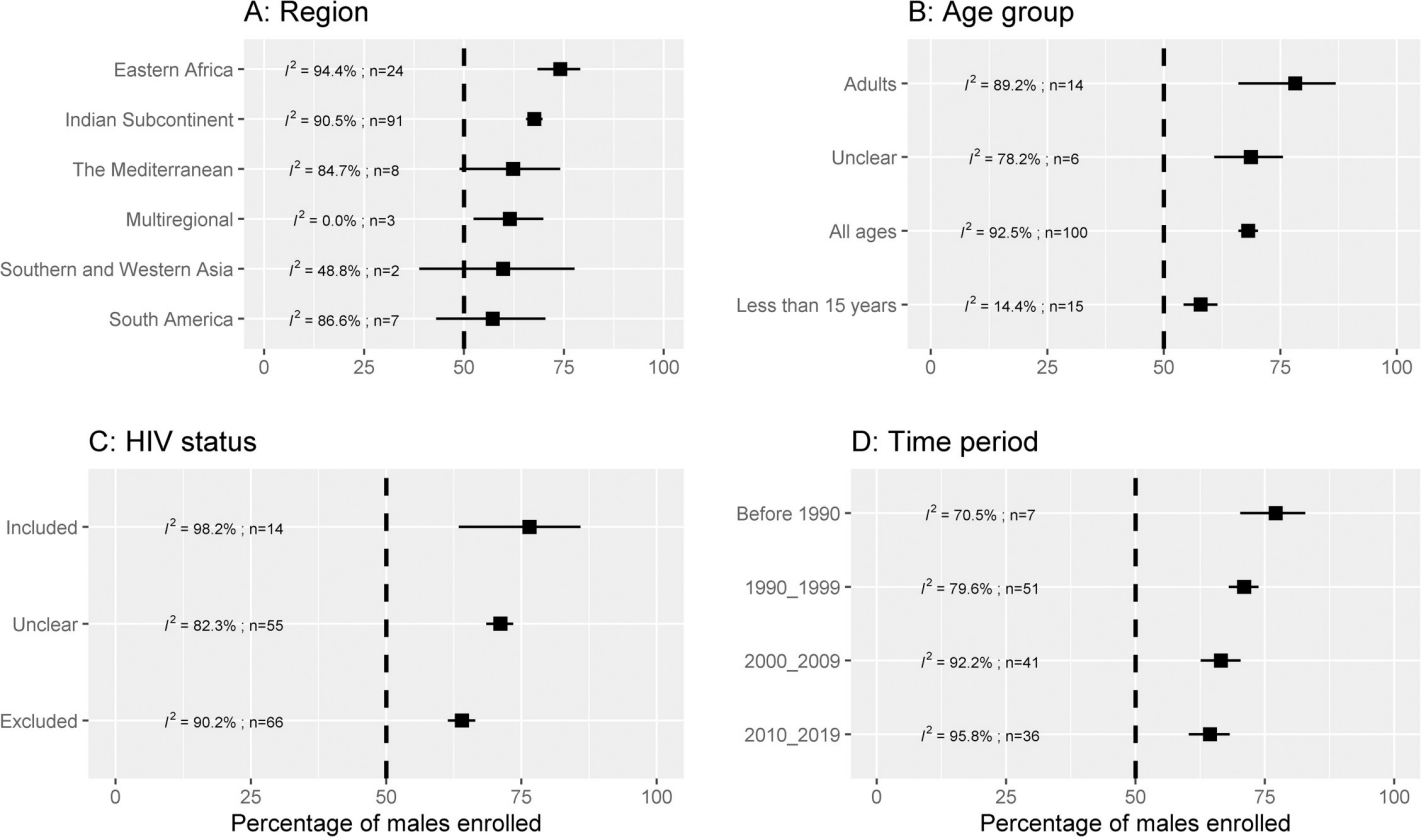
Percentage of males enrolled in VL clinical trials. *I*^*2*^ is a measure of heterogeneity (larger values indicate more heterogeneity); *n* is the number of studies combined for generating the pooled estimate. The vertical dotted line represents no difference in the enrolled proportion of males and females. The error bars are 95% confidence interval obtained from random effects meta-analysis of proportions.

In total, the 135 studies included in the review enrolled 32,177 patients, of whom 21,193 (65.9%) were males and 10,984 (34.1%) were females. From a random effects meta-analysis, the overall pooled estimate of male participants enrolled was 68.0% [95% confidence interval (CI): 65.9%–70.0%; 95% prediction interval (PI): 44.4%–85.0%; I^2^ = 92.6%]. After stratifying by region, the estimate was 67.6% [95% CI: 65.5%–69.7%; n = 91 trials; I^2^ = 90.5%; 24,218 patients] in studies conducted in the Indian sub-continent, 74.1% [95% CI: 68.4%–79.1%; n = 24 trials; I^2^ = 94.4%; 6,716 patients] in studies from Eastern Africa, 62.3% [95% CI: 48.9%–74.1%; n = 8 trials; I^2^ = 84.7%; 467 patients] in the Mediterranean region, and 57.2% [95% CI: 42.9%–70.4%; n = 7 trials; I^2^ = 86.6%; 607 patients] in Southern America (**Fig 2A**). There was large heterogeneity in results (I^2^ = 92.6%) and further sub-group meta-analyses were carried out to explore the potential sources of heterogeneity.

### Distribution by age-group

Patients of all ages were recruited in 100 (74.1%) studies, only children less than 15 years in 15 (11.1%) studies, only adults in 14 (10.4%) studies, and the age-distribution was not clear in 6 (4.4%). In studies that enrolled only children less than 15 years, the pooled estimate of male patients enrolled was 57.9% [95% CI: 54.2%–61.5%; n = 15 trials; I^2^ = 14.4%; 920 patients]. The corresponding estimate in studies enrolling only adults was 78.2% [95% CI: 66.0%–86.9%; n = 14 trials; I^2^ = 89.2%; 666 patients], and 68.1% [95% CI: 65.9%–70.0%; n = 100 trials, I^2^ = 92.5%; 29,649 patients] in studies that enrolled patients of all ages (**Fig 2B**). The estimates were further stratified by region and are presented in Table 1.

**Table 1:** Percentage of male patients enrolled in clinical studies estimated using random effects meta-analysis.

	Indian sub-continent				Eastern Africa			
Age range	*k*	*n*	Males enrolled [95% CI]	I^2^	*k*	*n*	Males enrolled [95% CI]	I^2^
Less than 15 years	6	418	61.2% [56.5–65.8]	0.0%	2	59	71.2% [58.4–81.3]	0.0%
Adults	6	295	70.4% [62.7–77.1]	17.9%	2	81	97.5% [90.7–99.4]	0.0%
All ages	73	22563	67.7% [65.2–69.9]	92.3%	20	6576	71.5% [66.3–76.2]	92.7%
Not clear	6	942	68.7% [60.8–75.6]	78.2%	-	-	-	-
**Time period**								
Before 1990	4	1257	80.1% [74.0–85.1]	70.2%	3	99	69.7% [60.0–77.9]	0.0%
1990 through 1999	35	5472	72.8% [70.2–75.2]	68.7%	7	501	68.1% [54.5–79.1]	85.1%
2000 through 2009	28	7188	65.7% [61.5–69.7]	91.7%	6	1349	72.7% [62.1–81.3]	92.6%
On or after 2010	24	10301	61.3% [59.8–62.7]	45.1%	8	4767	79.9% [71.6–86.2]	95.7%
**Inclusion of pregnant and lactating mothers**								
Included	6	3600	68.9% [60.6–76.2]	94.6%	5	3502	63.9% [56.5–70.7]	76.3%
Excluded	41	10570	65.1% [63.1–67.0]	70.7%	10	1804	79.1% [72.5–84.4]	83.1%
Unclear	44	10048	69.5% [65.8–72.9]	92.2%	9	1410	73.2% [62.2–81.9]	92.7%
**Inclusion of women of child bearing age**								
Excluded	-	-	-		1	151	81.5% [74.5–86.9]	-
Included	5	1839	70.8% [61.5–78.6]	91.8%	5	3502	63.9% [56.5–70.7]	76.3%
Conditional inclusion upon agreeing to contraception usage	13	6365	63.1% [60.8–65.3]	59.6%	1	58	98.3% [88.7–99.8]	-
Unclear	73	16014	68.2% [65.6–70.6]	89.7%	17	3005	73.9% [68.1–79.0]	88.9%
**Inclusion of HIV co-infected patients**								
Included	1	120	61.7% [52.7–69.9]		7	4706	83.4% [68.2–92.2]	98.8%
Excluded	50	16172	63.5% [60.8–66.0]	90.0%	5	444	79.5% [75.5–83.0]	0.0%
Not clear	40	7926	72.9% [70.2–75.4]	81.0%	12	1566	65.4% [59.8–70.7]	72.7%
**FDA risk category (pregnant and lactating women) of the drugs studied**								
Category B drugs ^**a**^	38	8342	68.5% [65.7–71.3]	82.7%	3	237	67.5% [51.2–80.5]	81.7%
Category C drugs ^**a**^	28	6594	69.8% [64.9–74.3]	93.5%	15	6094	71.7% [65.3–77.4]	94.6%
Category D drugs ^**a**^	19	8503	62.4% [60.9–63.9]	39.8%	3	239	88.4% [66.3–96.7]	86.0%
Unclear/Unassigned ^**a**^	6	779	64.8% [58.3–70.8]	38.1%	3	146	76.0% [68.4–82.3]	0.0%
**Study design**								
Randomised	41	9314	69.4% [66.8–71.8]	82.7%	8	1750	78.9% [67.9–86.8]	94.0%
Non-randomised	43	13202	65.6% [63.1–68.0]	84.2%	14	4848	71.0% [63.6–77.5]	93.2%
Unclear	7	1702	65.7% [49.7–78.7]	96.7%	2	118	74.6% [66.0–81.6]	0.0%
**Overal**l (adjusted for small study-effects)^b^	91	24218	67.6% [65.5–69.7] (62.5% [59.9–65.0])	90.5%	24	6716	74.1% [68.4–79.1] (67.1% [60.7–72.9])	94.4%

*k* = number of studies combined; n = total number of patients; CI = confidence interval

^a^ Category B drugs include amphotericin B deoxycholate and liposomal amphotericin B; Category C drugs include pentavalent antimony and pentamidine; Category D drugs include miltefosine. The categories were extracted from published literature (reviewed in [38, 39]). In multi-armed trials, the study is assigned the worst of the known categories.

^b^ Small study effects were evaluated using linear regression test for funnel plot asymmetry (*P* = 0.128 for Eastern Africa, *P* = 0.008 for Indian sub-continent, *P* = 0.0285 for overall dataset). Bias-adjusted estimate was derived using trim-and-fill method with an estimate of 63.5% [95% CI: 61.1%–65.7%] obtained for overall dataset (135 studies).

### Exclusion of pregnant and lactating women and women of childbearing age

**Fig 3:**
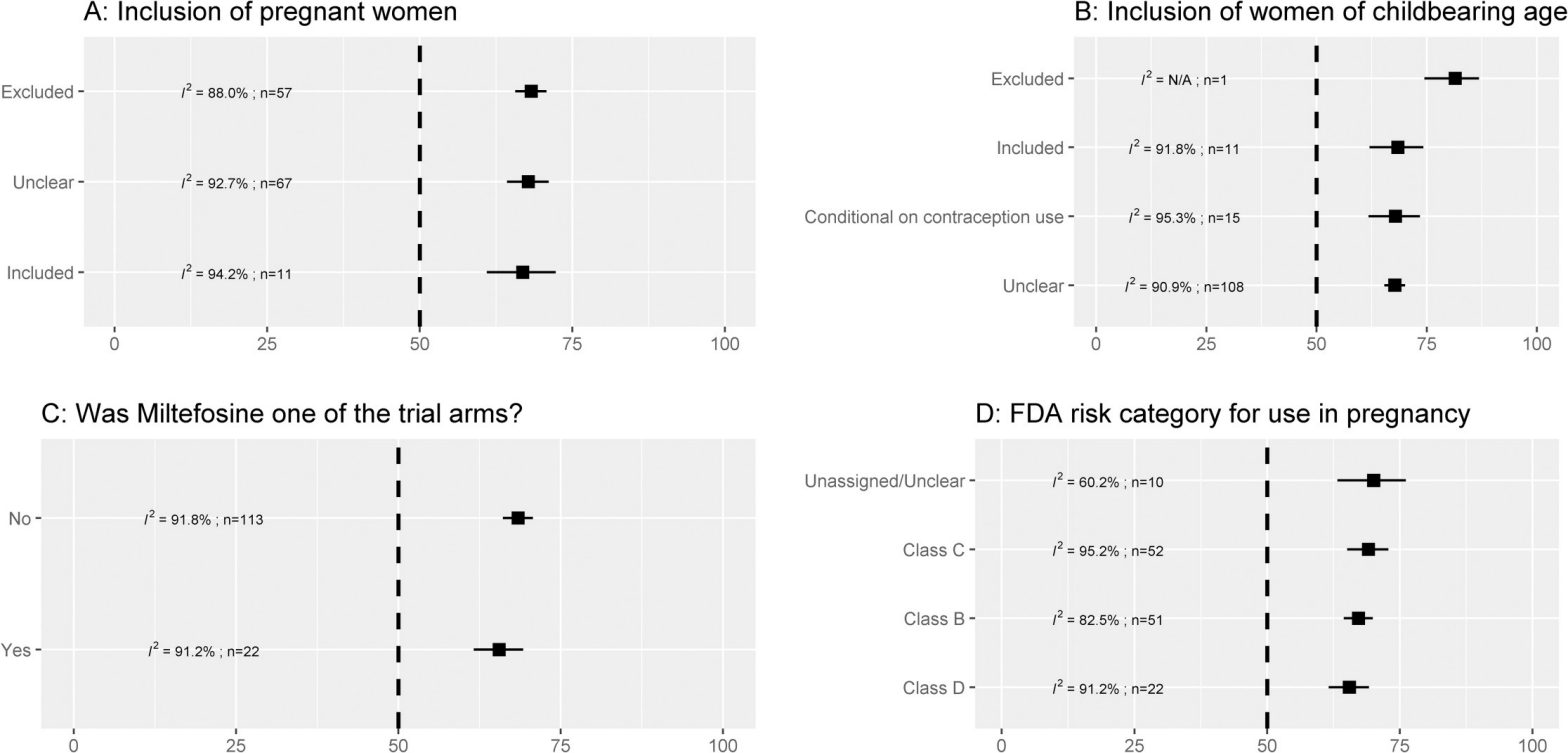
Percentage of males enrolled by pregnancy-related inclusion criteria. *I*^*2*^ is a measure of heterogeneity (larger values indicate more heterogeneity); *n* is the number of studies combined for generating the pooled estimate. The vertical dotted line represents no difference in the enrolled proportion of males and females. The error bars are 95% confidence interval obtained from random effects meta-analysis of proportions.

In 57 studies which excluded pregnant women, the pooled estimate of male patients enrolled was 68.3% [95% CI: 65.6%–70.8%; I^2^ = 88.0%] and in 11 studies which included pregnant women, the corresponding estimate was: 66.9% [95% CI: 60.9%–72.3%; I^2^ = 94.2%] (**Fig 3A**). In one study that explicitly stated excluding women of childbearing age, the proportion of males enrolled was 81.5% [42]. In studies that enrolled female participants conditional on contraception use for the treatment and follow-up period, the estimated proportion of males enrolled was: 67.9% [95% CI: 61.8%–73.5%; I^2^ = 95.3%; 15 trials] (**Fig 3B**). The proportion of males enrolled in studies which investigated the efficacy of miltefosine was 65.5% [95% CI: 61.6%–69.2%; I^2^ = 91.2%; 22 trials] and this was 68.5% [95% CI: 66.1%–70.7%; I^2^ = 91.8%; 113 trials] in studies which studied other drug regimens (**Fig 3C**). Overall, there were no differences in the proportion of males enrolled by the FDA drug category for use in pregnant and lactating women **(Fig 3D).** However, when stratified by region, the proportion of males enrolled was higher among drugs classed as FDA category C and D in studies conducted in Eastern Africa. In contrast, such differences were not observed in the Indian sub-continent (**Table 1**).

### Distribution in studies enrolling patients with HIV co-infections

Patients with HIV co-infections were included in 14 (10.4%) studies, excluded in 66 (48.9%), and not clear in remaining 55 (40.7%) studies. The estimate of male participants enrolled in studies that allowed enrolment of patients with HIV co-infections was 76.5% [95% CI: 63.4%–85.9%; I^2^ = 98.2%; n = 14 trials; 5,123 patients]. In 66 trials which excluded patients with HIV co-infections, the pooled estimate was 64.0% [95% CI: 61.4%–66.5%; I^2^ = 90.2%; 17,500 patients]. From the remaining 55 studies, where the HIV status of enrolled participants was not clear, the estimate was 71.1% [95% CI: 68.5%–73.5%; I^2^ = 82.3%; 9,554 patients] (**Fig 2C**). Further estimates stratified by region are presented in **Table 1**.

### Time-trends

**Fig 4:**
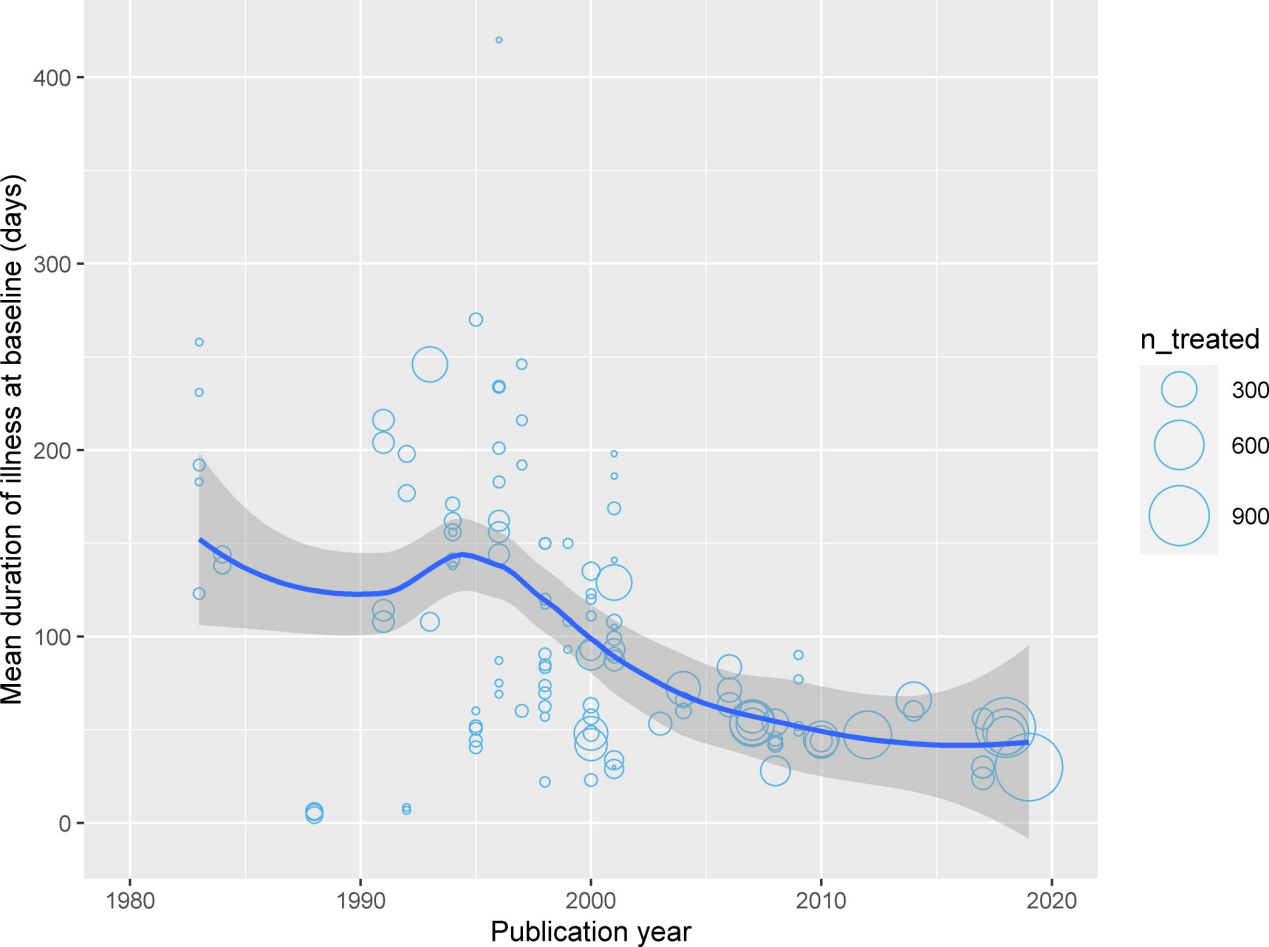
Mean duration of illness at presentation, days. The trend line is a lowess smoother and the confidence band is associated 95% confidence interval. Data available from 59 studies (13,164 patients) from a total of 132 study arms. Each circle represents a study arm and the size of the bubble is proportional to the number of patients enrolled in the study arm. The duration of illness was reported as median in 8 study arms and as a mean in 128 study arms (see S1 Data).

In studies published prior to the 1990s, the pooled estimate of males enrolled was 77.1% [95% CI: 70.2%–82.8%; I^2^ = 70.5%; n = 7 trials; 1,356 patients]. The corresponding estimates were 71.0% [95% CI: 68.0%–73.8%; I^2^ = 79.6%; n = 51 trials; 6,363 patients] in studies published from 1990 through 1999, 66.5% [95% CI: 62.6%–70.3%; I^2^ = 92.2%; n = 41 trials; 8,847 patients] in studies published from 2000 through 2009, and 64.4% [95% CI: 60.3%–68.2%; I^2^ = 95.8%; n = 36 trials; 15,611 patients] in articles published on or after 2010 (**Fig 2D**). In studies conducted on or after 2010 in the Indian sub-continent, the proportion of males enrolled was 61.3% [95% CI: 59.8%–62.7%; n = 24 trials; 10,301 patients] while the corresponding estimate in studies conducted in Eastern Africa was 79.9% [95% CI: 71.6%–86.2%; n = 8 trials; 4,767 patients] (Table 1). Data on mean duration of illness at presentation were available from 59 studies (13,164 patients) and a negative correlation between mean illness duration and publication year was observed (Pearson’s correlation coefficient: -0.42 [95% CI: -0.55 to -0.26]) (Fig 4).

### Impact of study design and assessment of risk of bias

The proportion of males enrolled in randomised studies was 70.9% [95% CI: 68.1%–73.5%; I^2^ = 89.3%; n = 53 trials; 11,643 patients]; 65.5% [95% CI: 62.7%–68.2%; I^2^ = 91.6%; n = 71 trials; 18,663 patients] in non-randomised studies, and 65.9% [95% CI: 54.6%–75.6%; I^2^ = 94.0%; n = 11 trials; 1,871 patients] in studies with unclear randomisation status. The proportion of male patients enrolled was 70.5% [65.9%–74.7%; I^2^ = 85.8%; 3,838 patients] in 23 Phase II-III studies, 62.2% [58.9%–65.4%; I^2^ = 83.6%; 7,254 patients] in 6 Phase IV studies, and 67.9% [65.4%–70.3%; I^2^ = 85.8%; 21,085 patients] in 106 studies with unclear phase information. Further details stratified by regions are presented in Table 1.

The risk of bias assessment in studies included in this review is presented in S1 Data and S1 and S2 Tables). One (1.9%) of the randomised studies was considered to be at a high risk of bias for random sequence generation domain, none were at high risk bias for allocation concealment, and 39 (73.5%) were at high risk of bias on the blinding domain (S1 Table). Of the 82 studies that were either single-armed or non-randomised or with unclear randomisation status, 13 (15.8%) were considered to be at a high risk of bias due to confounding, 17 (20.7%) at high risk of bias in selection of participants, and 15 (18.3%) were at a high risk of bias on the blinding domain (S2 Table). The estimates of males enrolled after stratifying by the risk of bias status did not indicate a substantial difference in the estimates (S1 and S2 Tables).

## Discussion

We identified 135 clinical treatment trials of VL published in the past 40 years (1980–2019) enrolling 32,177 patients and found that, overall, more than two-thirds of the enrolled patients were males. The result mirrors the observation that, in an endemic situation, VL in general appears to be more reported among males than females, whether due to behavioural or biological propensity [7, 9, 28](See S3 Table). Such observation of male preponderance has also been reported for the cutaneous form of leishmaniasis [14, 43, 44] and also in a post-Kala-azar dermal leishmaniasis study [45]. South Sudan is a notable exception with 57% of the 3,474 cases reported to the WHO in 2015 were females [46, 47].

The gender imbalance observed in the studies included in this review, however, was not homogenously distributed across and within time-period, geographical regions, or age-bands. A decreasing proportion of males were enrolled over time; with males being 3.4 times more likely to be included in studies published before the 1990s, 2.4-times, 1.9-times, and 1.8-times more likely in studies published through the 1990s, 2000s, and 2010s respectively. Such gradual increase in females enrolled over time might be due to a wider access to health facilities, diagnostic availability or improved female literacy over time [48]. The observation of a progressive reduction in mean illness duration at clinical presentation over time possibly indicates that improved access to care or awareness could be a possible explanation. However, the breakdown on duration of illness at presentation by gender was not available. While this overall trend was also observed in studies conducted in the Indian sub-continent, an opposite trend was observed in studies conducted in Eastern Africa with 3.9-times more males enrolled in studies conducted in the 2010s compared to 2.7-times more likely in studies conducted in the 2000s, and 2.3-times more likely in studies published in the 1990s. One possible explanation for such regional differences could be the increased surveillance activities in the Indian sub-continent due to the ongoing elimination programme (2005 onwards) and the inclusion of female community health workers within the programme [49, 50]. For example, in a PKDL study in India, there was an absence of gender bias in data collected from recent active surveillance (door-to-door visit) programme from 2015 onwards whereas data from a passive surveillance (2003 onwards) indicated predominance of males [51]. It is interesting to note that the (few) trials labelled as phase IV (i.e. post-registration), also report having enrolled 1.6-times more males than females.

The observed regional differences could also be due to the heterogeneity in the patient population studied and may also reflect different levels of endemicity, exposure, and/or behaviours. For example, the gender difference was more marked in studies that did not exclude patients co-infected with HIV, which had 3.3-times as many males, compared to 1.8-times as many in studies which clearly excluded HIV-infected subjects. In sub-Saharan Africa, the prevalence of HIV is higher among females including in Kenya and Ethiopia—where VL is highly endemic [52]. However, only 5.7% of the cases from five East African studies in HIV co-infected patients were females, and the reasons for such a lower proportion of females enrolled is not clear and requires further investigation.

The ratio of males to females was closer to parity in studies that enrolled only paediatric patients under 15 years of age (and no/little heterogeneity), while males were 3.6-times more represented in adult-only trials (substantial heterogeneity in results). Such observation that the gender-specific differences are attenuated among young and prepubescent children less than 15 years is consistent with several published data from the programmatic settings of VL in Brazil [26, 53–56] and India [57]; and such observation has also been noted for several other infectious diseases [58–61]. However, in the vast majority of studies included in this review, patients of all age group were included (>80% enrolled patients of all ages) and gender-specific breakdown by age group were not available thus limiting the robustness of this finding (heterogeneity was very high).

A major limitation of this review is that distribution of caseloads derived from efficacy studies might not be a reflection of the true distribution of caseloads in the community. There was also a large heterogeneity in the results, and the analysis was affected by unavailability of the breakdown of gender by age-group. Sensitivity analysis carried out by study design (randomisation status) or the risk of bias status on different domains did not indicate a substantial difference in the enrolled proportion of males (S1 and S2 Tables). We were also not able to identify whether the observed disparity could have been artificially maintained by the exclusion of pregnant women and women of childbearing age, drug regimens studied, due to gender-specific behavioural differences or biological causes. Some of these limitations can be addressed if individual participant data (IPD) from clinical studies are available. The Infectious Diseases Data Observatory (IDDO) is currently working towards collating and standardising VL datasets from as many as possible retrievable clinical studies included in this systematic review in order to conduct IPD meta-analyses [34].

In conclusion, VL treatment guidelines are informed by a knowledge of treatment outcomes from a population that is heavily skewed towards adult males, overwhelmingly from high-burden countries of the Indian sub-continent and Eastern Africa, though with some notable regional variations and substantial heterogeneity. Without individual-participant level meta-analysis, and possibly access to screening logs documenting reasons for excluding patients, it is not possible to understand if differential treatment outcomes can be expected in males and females, and what the underlying factors might be driving such observed disparity.

## Supporting information

S1 Text PRISMA checklist. (DOCX)

S1 Data Description of the studies included and data used for analysis. (XLSX)

S1 Table Estimates of males enrolled by risk of bias status in randomised studies. (DOCX)

S2 Table Estimates of males enrolled by risk of bias status in non-randomised or studies with unclear randomisation status. (DOCX)

S3 Table Explanations on observed differences in gender distribution in studies included. (DOCX)
