# Supplementary material for: Gender disparity in cases enrolled in clinical trials of visceral leishmaniasis: A systematic review and meta-analysis
Source: PLoS Negl Trop Dis. 2021 Mar 16;15(3):e0009204. doi: 10.1371/journal.pntd.0009204 (PMC7963105; doi:10.1371/journal.pntd.0009204)
Supplement: S1 Table — (DOCX) [file pntd.0009204.s003.docx]

**S1 Table. Estimates of males enrolled by risk of bias status in randomised studies**

| **Domain** | ***k*** | **Estimate [95% CI] from**  **random effects meta-analysis** | **I^2^** |
| --- | --- | --- | --- |
| **Random sequence generation** |  |  |  |
| High risk of bias | 1 | 73.9% [67.9–79.2] | - |
| Low risk of bias | 32 | 69.1% [65.1–72.7] | 92.5% |
| Unclear risk of bias | 20 | 73.8% [70.2–77.0] | 72.0% |
| **Allocation concealment** |  |  |  |
| High risk of bias | - | - | - |
| Low risk of bias | 26 | 68.9% [64.3–73.2] | 92.9% |
| Unclear risk of bias | 27 | 72.8% [69.6–75.7] | 79.6% |
| **Blinding of participants and personnel** |  |  |  |
| High risk of bias | 39 | 70.5% [67.0–73.7] | 91.5% |
| Low risk of bias | 2 | 69.7% [54.0–81.8] | 80.5% |
| Unclear risk of bias | 12 | 72.5% [68.1–76.5] | 72.4% |

*k*= number of studies combined; CI = confidence interval; *I^2^* is a measure of heterogeneity – larger values indicates more heterogeneity
