# Supplementary material for: Gender disparity in cases enrolled in clinical trials of visceral leishmaniasis: A systematic review and meta-analysis
Source: PLoS Negl Trop Dis. 2021 Mar 16;15(3):e0009204. doi: 10.1371/journal.pntd.0009204 (PMC7963105; doi:10.1371/journal.pntd.0009204)
Supplement: S2 Table — (DOCX) [file pntd.0009204.s004.docx]

**S2 Table. Estimates of males enrolled by risk of bias status in non-randomised or studies with unknown randomisation status**

| **Domain** | ***k*** | **Estimate [95% CI] from**  **random effects meta-analysis** | **I^2^** |
| --- | --- | --- | --- |
| **Bias due to confounding** |  |  |  |
| High risk of bias | 13 | 62.4% [51.2–72.3] | 97.2% |
| Moderate risk of bias | 11 | 66.8% [57.4–75.0] | 85.6% |
| Low risk of bias | 4 | 69.9% [62.8–76.2] | 83.0% |
| Unclear risk of bias | 7 | 64.5% [56.1– 72.0] | 37.6% |
| Single arm studies | 47 | 65.7 % [62.9–68.4] | 85.6% |
| **Bias in participant selection** |  |  |  |
| High risk of bias | 17 | 68.6% [61.2–75.2] | 96.0% |
| Moderate risk of bias | 12 | 62.6% [60.5–64.6] | 0.0% |
| Low risk of bias | 53 | 64.5% [60.9–67.9] | 91.9% |
| Unclear risk of bias | - | - | - |
| **Blinding of participants and personnel** |  |  |  |
| High risk of bias | 15 | 67.4% [60.9–73.2] | 93.6% |
| Moderate risk of bias | 1 | 73.2% [63.5–81.1] | - |
| Low risk of bias | - | - | - |
| Unclear risk of bias | 19 | 62.5% [53.9–70.3] | 93.3% |
| Single arm studies | 47 | 65.7% [62.8–68.4] | 85.6% |

*k*= number of studies combined; CI = confidence interval; *I^2^* is a measure of heterogeneity – larger values indicates more heterogeneity
