# Supplementary material for: Gender disparity in cases enrolled in clinical trials of visceral leishmaniasis: A systematic review and meta-analysis
Source: PLoS Negl Trop Dis. 2021 Mar 16;15(3):e0009204. doi: 10.1371/journal.pntd.0009204 (PMC7963105; doi:10.1371/journal.pntd.0009204)
Supplement: S3 Table — (DOCX) [file pntd.0009204.s005.docx]

**S3 Table. Explanations on observed differences in gender distribution in studies included**

| **Study** | **Description from the manuscript** |
| --- | --- |
| Thakur-1984a^1^ | "Most of the population are either farmers or farm labourers and most farmers have cowsheds, or bathans, where they keep cattle. These cowsheds have been found heavily infested with sand-flies. The cattle are looked after by the male members of families of rich farmers and higher castes and by farm labourers. Children often play in the bathans during the day but do not sleep there at night. Females of poor families also tend the cattle". "In most houses females and children slept inside and males on the verandah" |
| Nyakundi-1994^2^ | “Most of the cases are male as they spend most of the time outdoors where they get bitten by sand flies” |
| Mishra-1994^3^ | “The preponderance of male patients in our series was striking. This was probably because the Indian female is at all times better covered with clothes than the male and hence less prone to vector bite.” |
| Thakur-1999^4^ | “The greater proportion of males than females (ratio 4.2:1) may reflect the facts that AMB treatment is expensive (US$600 each patient), and that many females with untreated visceral leishmaniasis were not brought to this tertiary level of health care and died” |
| Haidar-2001^5^ | “A significant variation was not expected due to the similar rate of exposure in children regardless of the sex difference.” |
| Thakur-2001^6^ | An observation of more females than males were made in this study, which is in contrast to reports from tertiary care level of medical care in Patna, where males outnumbered females in far greater ratio. We had a similar experience of field study when the ratio was almost equal. Napier also observed that at the field level, the incidence of both sexes are equal. Possibly males are preferred for more costly treatment at the tertiary level, besides the fact that more males visit the tertiary care facility. |

1. Thakur CP. Epidemiological, clinical and therapeutic features of Bihar kala-azar (including post kala-azar dermal leishmaniasis). *Trans R Soc Trop Med Hyg*. 1984;78(3):391-398. doi:10.1016/0035-9203(84)90131-7

2. Nyakundi P, Wasunna K, Rashid JR, et al. Is one year follow up justified in Kala Azar post treatment. *East Afr Med J*. 1994;71(7).

3. Mishra M, Biswas UK, Jha AM, Khan AB. Amphotericin versus sodium stibogluconate in first-line treatment of Indian kala-azar. *Lancet*. 1994;344(8937):1599-1600. doi:10.1016/S0140-6736(94)90406-5

4. Thakur CP, Singh RK, Hassan SM, Kumar R, Narain S, Kumar A. Amphotericin B deoxycholate treatment of visceral leishmaniasis with newer modes of administration and precautions: A study of 938 cases. *Trans R Soc Trop Med Hyg*. 1999;93(3):319-323. doi:10.1016/S0035-9203(99)90037-8

5. Haider NA, Diab A-BL, EL-Sheikh AM. Visceral Leishmaniasis in children in the Yemen. *Saudi Med J*. 1990;11(2):99-104.

6. Thakur CP, Ahmed S. Observations on amphotericin B treatment of kala-azar given in a rural set up in Bihar, India. *Indian J Med Res*. 2001;113(JAN.):14-18.
